# Bone Mineral Density in Black South African Women Newly Diagnosed With Breast Cancer Living With and Without HIV

**DOI:** 10.1200/GO-25-00122

**Published:** 2025-06-18

**Authors:** Lindor Qunaj, Maureen Joffe, Alfred I. Neugut, Lisa K. Micklesfield

**Affiliations:** ^1^ Division of Hematology/Oncology, Department of Medicine and Herbert Irving Comprehensive Cancer Center, Columbia University Irving Medical Center, New York, NY; ^2^ Strengthening Oncology Services Research Unit, Faculty of Health Sciences, University of the Witwatersrand, Johannesburg, South Africa; ^3^ Department of Epidemiology, Mailman School of Public Health, Columbia University, New York, NY; ^4^ SAMRC/Wits Developmental Pathways for Health Research Unit, Faculty of Health Sciences, University of the Witwatersrand, Johannesburg, South Africa

## Abstract

**PURPOSE:**

Worsening bone mineral density (BMD)—and the corresponding increase in osteoporotic fractures—is an important and well-established source of morbidity and mortality in women receiving treatment of breast cancer, as well as those living with HIV. However, there are comparatively few reports on pretreatment bone health in women newly diagnosed with breast cancer, especially in predominantly Black populations, across sub-Saharan Africa (SSA), and among individuals living with HIV. Therefore, we sought to characterize bone health in a cohort of Black South African women with and without HIV before the initiation of systemic breast cancer therapy, in particular chemotherapy and/or aromatase inhibitors.

**METHODS:**

Building on the South African Breast Cancer and HIV Outcomes study, we recruited consecutive women newly diagnosed with stage I-III breast cancer who were to start systemic cancer therapy at the Chris Hani Baragwanath Academic Hospital in Soweto, Johannesburg, between June 2021 and August 2024. In addition to collecting extensive demographic and clinical information, we conducted dual energy X-ray absorptiometry (DXA) scans on each patient to measure BMD of the lumbar spine, femoral neck, and total hip.

**RESULTS:**

We enrolled a total of 378 women, 32.3% of whom (n = 122) were living with HIV. Among women aged 50 years and older (n = 156), 64.1% had osteopenia or osteoporosis; HIV infection and vitamin D insufficiency/deficiency—but no breast cancer characteristics—were associated with a higher risk of osteoporosis. By contrast, 3.6% of women younger than 50 years had BMD below the expected range for age.

**CONCLUSION:**

Especially in low-resource clinical settings, such as public hospitals in SSA, understanding which women are at highest risk of osteoporosis and fragility fracture before the initiation of breast cancer systemic therapy is critical. Our study provides a foundation for identifying relevant risk factors and ultimately designing interventional studies that target high-risk women for intensified osteoporosis screening and management.

## INTRODUCTION

The deleterious effects of breast cancer treatment on bone mineral density (BMD) are well established. In particular, women who have received treatment of breast cancer are at significantly increased risk of osteopenia or osteoporosis, as defined by lower than expected BMD, when compared with healthy women.^1^ Higher rates of osteoporosis translate to more skeletal fractures; one meta-analysis of four studies that included 127,000 patients estimated that women with breast cancer were at a 35% increased risk of osteoporotic fractures compared with women without cancer (risk ratio, 1.35 [95% CI, 1.29 to 1.42]), excluding pathologic fractures due to skeletal metastases.^2^ Much of this bone loss—and its subsequent complications—has been attributed to two classes of drugs used in the management of hormone receptor–positive (HR+) breast cancer: chemotherapy and aromatase inhibitors (AIs). Both of these treatments lead to a state of estrogen deprivation, which is a major risk factor for the development of osteoporosis; chemotherapy exerts its effect by inducing primary ovarian failure, which can lead to either transient amenorrhea or complete menopause, while AIs block the enzymatic production of estrogen.^3,4^ These impacts may lead to significant morbidity as demonstrated in the randomized ATAC trial, which established the AI anastrozole as a standard-of-care adjuvant therapy in localized HR+ breast cancer: Across a combined >6,200 patients, the incidence rate of fractures was 55% greater in women receiving anastrozole compared with tamoxifen.^5^

**CONTEXT**

**Key Objective**
To determine the baseline prevalence of osteopenia and osteoporosis in a cohort of Black women newly diagnosed with breast cancer, and to identify the clinical risk factors associated with osteoporosis in this population, including comorbid HIV infection.
**Knowledge Generated**
Nearly two thirds (64.1%) of 378 women who were enrolled at a large academic teaching hospital in Johannesburg, South Africa, had osteopenia or osteoporosis at the time of their breast cancer diagnosis. In women older than 50 years, HIV infection and low vitamin D levels were associated with higher odds of having osteoporosis, whereas obesity was associated with lower odds of osteoporosis.
**Relevance**
In clinical settings where radiographic bone mineral density assessment is not readily available and HIV prevalence is high, our study provides a foundation for more precise selection of women at highest risk of osteoporosis before the initiation of breast cancer therapy, as well as the subsequent design of interventional trials aimed at reducing this important cause of morbidity and mortality.

Similar to women treated for breast cancer, those living with HIV experience higher rates of osteoporosis and its morbid complications, with studies from South Africa and Zimbabwe demonstrating a two- to five-fold higher osteoporosis prevalence in individuals with HIV compared to those without.^6-9^ While HIV infection itself has a deleterious impact on bone metabolism, the largest contribution to comorbid osteoporosis among individuals living with HIV is the initiation of combined antiretroviral therapy (ART), which has been associated with a 2%-6% decline in BMD over the first 2 years of use.^10^ Bone loss is especially well established as an adverse effect of nucleotide reverse transcriptase inhibitors, such as tenofovir, but other HIV drug categories including protease inhibitors have been shown to have a negative effect as well.^11^

Despite these clear links, the association between HIV and BMD specifically in the context of breast cancer has not been studied as extensively. Similarly, little is known about the factors associated with baseline BMD in women newly diagnosed with breast cancer but not yet on systemic therapy. Given the impact of HIV infection and ART on bone health, we hypothesize that women living with HIV will have lower BMD than women without HIV at the time of their breast cancer diagnosis, raising concerns for a higher risk of osteoporotic fractures after initiating chemotherapy and/or AIs.

To date, much of the research investigating bone health in patients with breast cancer has focused on White populations and/or high-income countries.^1,2,12^ The underrepresentation of Black women—especially those living in sub-Saharan Africa (SSA)—represents a key shortcoming with important public health implications. Recently, rapid economic development and corresponding increases in life expectancy across SSA have led to dramatic increases in the prevalence of noncommunicable diseases, including both breast cancer and osteoporosis.^13-16^ Moreover, HIV remains highly prevalent in the region, with as much as one third of young South African women living with this disease.^17^ Consistent with general population trends, women with HIV are living longer because of scientific advances in ART and other investments in HIV care throughout the region; as a result, HIV-related mortality has decreased, but the incidence of comorbid HIV with diseases of aging—such as cancer and osteoporosis—continues to rise.^18,19^

Acknowledging the link between breast cancer treatment and bone health, ASCO recommends baseline and then biennial BMD evaluations by dual energy X-ray absorptiometry (DXA) for all women with breast cancer treated with AIs.^20^ Guidelines from multiple international groups, such as the Infectious Diseases Society of America and European AIDS Clinical Society, also recommend enhanced screening for osteoporosis in individuals living with HIV.^21,22^ However, the availability of DXA scanners—as well as of bisphosphonates, a class of medications consistently demonstrated to reduce the risk of fragility fractures in patients with osteoporosis—is limited in many low-resource clinical settings internationally, including public hospitals in South Africa. Therefore, a better understanding of the burden of osteopenia and osteoporosis in Black women with and without HIV before the initiation of breast cancer therapy is essential to identify patients at highest risk of osteoporosis and related fractures. In turn, this population can be preferentially targeted in interventions designed to reduce bone-related morbidity and mortality.

To that end, the overarching objective of our current analysis was to identify the demographic and clinical characteristics, including variables related to HIV infection and breast cancer, associated with BMD at the time of breast cancer diagnosis. In doing so, we will provide valuable insights for health systems in low- and middle-income countries or any clinical setting where access to DXA machines and bisphosphonates is limited.

## METHODS

### Study Population

In this study, we built upon the ongoing South African Breast Cancer and HIV Outcomes (SABCHO) cohort, the detailed methods and objectives of which were published in 2017.^23^ Since then, the SABCHO team has successfully enrolled and followed over 5,000 women newly diagnosed with breast cancer who were recruited from six public hospitals across South Africa, reporting extensively on their cancer-related outcomes.^24-27^ In the current study, we aimed to measure BMD in a subcohort of women with stage I-III breast cancer, by performing DXA scans on each of them at the time of diagnosis, before the initiation of systemic cancer treatment. Institutional review boards at both Columbia University (protocol AAAQ1359) and University of the Witwatersrand (Wits; M200138) provided approval for the study, and we obtained informed consent from each participant in accordance with these approvals.

DXA, which is the gold standard for measurement of BMD and diagnosis of osteoporosis, was not universally available at all six South African hospitals in the SABCHO initiative. As a result, we recruited women exclusively at the Chris Hani Baragwanath Academic Hospital (Bara) for this study because a DXA machine is available at the SAMRC/Wits Developmental Pathways for Health Research Unit (DPHRU), situated on the Bara campus. Bara is a public tertiary care hospital located in Soweto, a low-income township within the city of Johannesburg where >98% of the 1.8 million residents are Black.

### Patient Population

All women older than 18 years with histologically proven stage I-III breast cancer (and no prior malignancies) diagnosed at Bara were eligible for enrollment. Prior receipt of any breast cancer–directed treatment (chemotherapy, radiation, and/or surgery) was an exclusion criterion. Women who consented to the study participated in an extensive intake interview with a study nurse, where they provided detailed information on their socioeconomic background, reproductive history (such as age at menarche, parity, and menopausal status), and medical history. Menopausal status was self-reported, with postmenopause defined as the absence of menstruation for 12 months or more, perimenopause as a lengthening menstrual cycle with or without vasomotor symptoms in the past year, and premenopause as ongoing menstruation with a standard cycle length of approximately 28 days. BMI, calculated as weight (in kilograms) divided by height (in meters) squared, was reported as a continuous variable and also categorized into normal weight (<25 kg/m^2^), overweight (25-29.9 kg/m^2^), or obese (≥30 kg/m^2^). The patient's breast cancer characteristics included histologic grade, HR status determined by immunohistochemistry, and stage; we did not exclude women on the basis of their tumor's histologic grade or HR status.

### Serologic Testing

Blood samples were collected on all women to obtain baseline serum 25-hydroxy vitamin D levels (in ng/mL) using the LIASION DiaSorin chemiluminescence assay. On the basis of the consensus statement from an international panel of experts convened in 2017, we categorized vitamin D levels as sufficient (≥20 ng/mL), insufficient (12-19.9 ng/mL), or deficient (<12 ng/mL).^28^ Participants were asked to self-report their HIV status, and confirmatory testing was performed through the South African National Health Laboratory Service using an enzyme-linked immunosorbent assay. Women living with HIV were offered infectious disease specialty care and had baseline testing of CD4 count (reported in cells/μL) and HIV viral load (copies/mL).

### BMD Reporting

We assessed BMD using a Hologic Discovery A DXA scanner (Hologic Inc, Bedford, MA; Apex software version 13.4.2:3; S/N 83145) located at the DPHRU. The instrument was operated by a trained technician, and scanner performance was monitored using standard manufacturer quality assurance and quality control protocols. Measurements of BMD expressed in grams per square centimeter (g/cm^2^) at the lumbar spine (LS), total hip (TH), and femoral neck (FN) were completed. For women aged 50 years and older, sex-specific reference data from White individuals in the National Health and Nutrition Examination Survey III were used to derive T-scores, according to recommendations from the International Society for Clinical Densitometry (ISCD), which recognize the absence of local reference data.^29^ We then categorized T-scores, defined as the number of standard deviations a patient's bone density is from the median expected for a healthy 30-year-old woman, based on the WHO's classification scheme: T-scores ranging from –1.0 to –2.5 represent osteopenia, <–2.5 osteoporosis, and >–1.0 normal BMD. For women younger than 50 years, the ISCD recommends the use of Z-scores, which compare the patient's BMD with women of the same age.^29^ We therefore categorized women younger than 50 years as having below expected range for age or normal BMD (Z-score <–2 and >–2, respectively).

### Statistical Analysis

We calculated means and medians for continuous variables, reporting the appropriate statistic depending on the skewness of data distributions. Student *t*-tests were performed to assess differences in means between individuals living with HIV and those without. For categorical variables, chi-squared tests were used to compare HIV groups. For women aged 50 years and older, we developed a multivariable logistic regression model to determine the odds of osteoporosis (defined as T-score of <–2.5 at any skeletal site) among patients with HIV compared with those without, adjusting for obesity, advanced age (>65 years), vitamin D insufficiency/deficiency, and breast cancer characteristics associated with poor prognosis (grade 3 histology and stage III disease). We replicated this model for women younger than 50 years to determine the relative odds of a Z-score below the expected range for age. All analyses were performed using Stata version 18.0 (Statacorp LLC, College Station, TX).

## RESULTS

### General Characteristics

**FIG 1:**
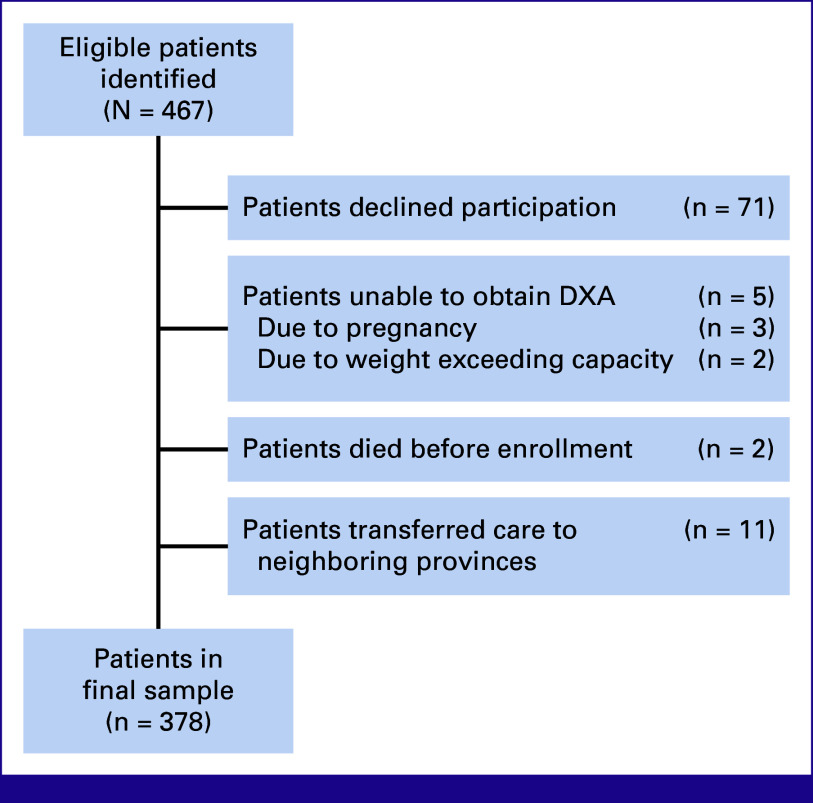
Summary of patient identification and enrollment into the BMD-focused SABCHO subcohort. BMD, bone mineral density; DXA, dual energy X-ray absorptiometry; SABCHO, South African Breast Cancer and HIV Outcomes.

Between June 1, 2021, and August 31, 2024, we identified 467 eligible patients newly diagnosed with stage I-III breast cancer. Of these, 378 (80.9%) consecutive consenting women were enrolled, with the remainder declining participation in the study, unable to safely undergo a DXA scan, dying before enrollment, or transferring their oncologic care to a clinic in a different province (Fig 1). Approximately one third (n = 122, 32.3%) of the women in our cohort were living with HIV, and among them, 95.9% were on ART and the median CD4 count was 630 cells/μL (Table 1). The mean age of the women in our cohort was 48.3 years, with no statistical difference between women living with and without HIV. Compared with women without HIV, women living with HIV were less likely to be obese (44.3% *v* 61.7%, *P* < .001) but more likely to smoke tobacco (8.2% *v* 2.7%, *P* = .016). The median BMI in the cohort, however, was 30.9 kg/m^2^ (IQR, 26.7-35.3), corresponding to obesity. Nearly half (46.6%) of the women were perimenopausal or postmenopausal, and 20.9% had either vitamin D deficiency or insufficiency.

**TABLE 1:** Demographic and Clinical Characteristics of Women Receiving Dual Energy X-Ray Absorptiometry Scans After Breast Cancer Diagnosis

Variable	Patients Living With HIV (n = 122)	Patients Without HIV (n = 256)	Full Cohort (n = 378)	*P*
Age, years, mean (SD)	47.2 (8.6)	48.9 (12.4)	48.3 (11.3)	.164
<50 years, No.(%)	78 (63.9)	144 (56.3)	222 (58.7)	
≥50 years, No. (%)	44 (36.1)	112 (43.8)	156 (41.3)	
BMI, median (IQR)	28.2 (24.9-33.6)	32.0 (27.8-36.4)	30.9 (26.7-35.3)	<.001
Normal weight (<25 kg/m^2^), No. (%)	32 (26.2)	25 (9.8)	57 (15.1)	
Overweight (25-29.9 kg/m^2^), No. (%)	36 (29.5)	73 (28.5)	109 (28.8)	
Obese (≥30 kg/m^2^), No. (%)	54 (44.3)	158 (61.7)	212 (56.1)	
Tobacco use, No. (%)				.016^a^
Smoker	10 (8.2)	7 (2.7)	17 (4.5)	
Nonsmoker	111 (91.0)	248 (96.9)	359 (95.0)	
Living with HIV				
Years since HIV diagnosis, median (IQR)	10 (4-15)			
On antiretroviral therapy,^b^ No. (%)	117 (95.9)			
CD4 count, cells/μL,^b^ median (IQR)	630 (432-871)			
HIV viral load, copies/mL,^b^ median (IQR)	0 (0-31)			
Reproductive history				
Age at menarche, years, mean (SD)	14.8 (2.1)	14.8 (2.0)	14.8 (2.0)	.856
Any prior pregnancy, No. (%)	113 (92.6)	241 (94.1)	354 (93.7)	.665^a^
Age at first pregnancy, years, median (IQR)	21 (18-25)	21 (19-24)	21 (19-25)	.893
Number of full-term pregnancies, median (IQR)	2 (2-3)	2 (2-3)	3 (2-3)	.430
Menopausal status, No. (%)				.501^a^
Premenopausal	68 (55.7)	132 (51.6)	200 (52.9)	
Perimenopausal	6 (4.9)	9 (3.5)	15 (4.0)	
Postmenopausal	47 (38.5)	114 (44.5)	161 (42.6)	
Age at menopause, years, median (IQR)	45 (40-50)	45 (42-50)	45 (41-50)	.570
Baseline vitamin D (25OH) levels, ng/mL,^b^ median (IQR)	35.2 (21.6-61.0)	32.3 (20.6-55.8)	33.1 (21.1-57.9)	.330
Sufficiency (≥20 ng/mL), No. (%)	98 (80.3)	201 (78.5)	299 (79.1)	
Insufficiency (12-19.9 ngl/mL), No. (%)	16 (13.1)	35 (13.7)	51 (13.5)	
Deficiency (<12 ng/mL), No. (%)	8 (6.6)	20 (7.8)	28 (7.4)	
Breast cancer stage at diagnosis, No. (%)				.966^a^
I	2 (1.6)	5 (2.0)	7 (1.9)	
II	43 (35.3)	92 (35.9)	135 (35.7)	
III	77 (63.1)	159 (62.1)	236 (62.4)	
Breast cancer receptor status, No. (%)				.781^a^
HR+^c^	89 (73.0)	184 (71.9)	273 (72.2)	
HR–/HER2+	11 (9.0)	19 (7.4)	30 (7.9)	
Triple negative (ER–/PR–/HER2–)	22 (18.0)	52 (20.3)	74 (19.6)	
Histologic grade, No. (%)				.909^a^
1	6 (4.9)	14 (5.5)	20 (5.3)	
2	60 (49.2)	118 (46.1)	178 (47.1)	
3	54 (44.3)	115 (44.9)	169 (44.7)	

Abbreviations: HER2, human epidermal growth factor receptor 2; HR, hormone receptor; SD, standard deviation.

^a^ *P* value calculated from chi-squared test; remainder are from two-sided *t*-tests of difference in means.

^b^ Measured at the time of breast cancer diagnosis.

^c^ Estrogen and/or progesterone receptor expression ≥1%.

Consistent with prior studies of breast cancer in SSA, as well as our own previously published findings in the SABCHO cohort, it is rare for women to present with stage I disease; 98.1% of women in this cohort were diagnosed at stage II or III.^25,30^ Similarly, 91.8% had moderately to poorly differentiated cancers, a histologic indication of clinically more aggressive disease. Across the entire cohort, 72.2% of women had HR+ tumors. There were no differences in the distribution of cancer stage, grade, or receptor status between women living with and without HIV.

### Baseline BMD Assessments

**FIG 2:**
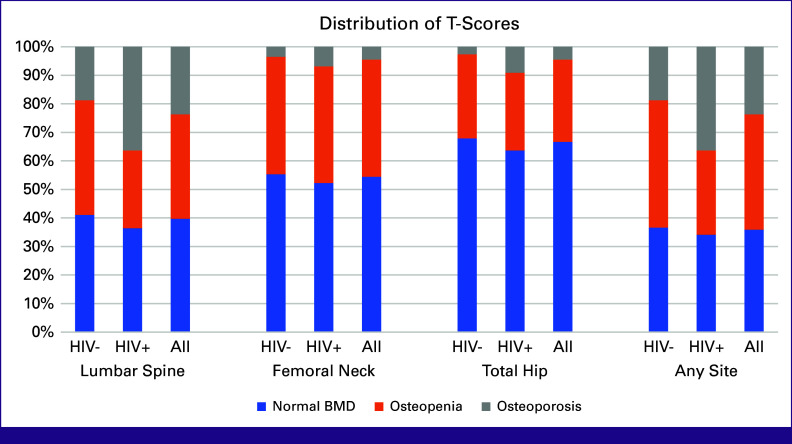
Prevalence of osteopenia and osteoporosis by skeletal site for women aged 50 years and older (n = 156). BMD, bone mineral density.

In women aged 50 years and older (n = 156), 64.1% had T-scores ≤–1 in at least one of the three skeletal sites; 23.7% met criteria for osteoporosis and 40.4% had osteopenia (Fig 2). The LS was the most commonly implicated skeletal site, with a mean T-score of –1.33 (standard deviation [SD], 1.65); it was also the only skeletal site where women living with HIV had a significantly different mean T-score compared with those without HIV (Table 2).

**TABLE 2:** T and Z-Scores at Each Skeletal Site by HIV Status

T-Scores (women aged 50 years and older)	Patients Living With HIV (n = 44), Mean (SD)	Patients Living Without HIV (n = 112), Mean (SD)	Full Cohort (n = 156), Mean (SD)	*P* ^a^
Skeletal site				
Lumbar spine	–1.69 (1.74)	–1.19 (1.60)	–1.33 (1.65)	.046
FN	–0.82 (1.26)	–0.76 (1.10)	–0.77 (1.15)	.387
TH	–0.60 (1.30)	–0.31 (1.27)	–0.39 (1.28)	.099
**Z-Scores (women younger than 50 years)**	**Patients Living With HIV (n = 78), Mean (SD)**	**Patients Living Without HIV (n = 144), Mean (SD)**	**Full Cohort (n = 222), Mean (SD)**	***P*** ^**a**^
Skeletal site				
Lumbar spine	–0.45 (0.91)	–0.15 (0.99)	–0.25 (0.97)	.012
FN	0.07 (0.85)	0.60 (1.08)	0.41 (1.03)	<.001
TH	–0.05 (0.85)	0.43 (0.94)	0.26 (0.94)	<.001
**Bone Mineral Density (g/cm^2^; all women)**	**Patients Living With HIV (n = 122), Mean (SD)**	**Patients Living Without HIV (n = 256), Mean (SD)**	**Full Cohort (n = 378), Mean (SD)**	***P*** ^**a**^
Skeletal site				
Lumbar spine	0.958 (0.155)	0.995 (0.154)	0.983 (0.155)	.014
FN	0.822 (0.122)	0.860 (0.140)	0.848 (0.136)	.005
TH	0.927 (0.126)	0.974 (0.136)	0.959 (0.134)	.001

Abbreviations: FN, femoral neck; TH, total hip.

^a^ Calculated from one-sided *t*-test for difference in means between patients living with HIV and those living without HIV.

Women aged 65 years and older were more likely to have osteopenia or osteoporosis than those aged 50-64 years (*P* = .002). The prevalences of osteopenia and osteoporosis in the ≥65-year age cohort (n = 35) were 54.3% and 31.4% compared with 36.4% and 21.5%, respectively, in the 50-64-year age cohort (n = 121).

In women younger than 50 years (n = 222), 3.6% had a Z-score below the expected range for age. Across both women with HIV and those without, the skeletal site with the lowest mean Z-score was the LS (–0.25, SD, 0.97), and the FN had the highest (0.41, SD, 1.03). In univariable analysis, women living with HIV had significantly lower Z-scores at the LS, FN, and TH compared with those without HIV. When comparing BMD across women of all ages in our cohort using unadjusted values (in g/cm^2^, rather than as standardized T and Z-scores), those living with HIV had lower BMD at each of the examined skeletal sites compared with those without HIV.

### Regression Analyses

When controlling for the presence of concurrent obesity, older age (>65 years), grade 3 histology, and stage III breast cancer, the odds of having osteoporosis at any site were 3.20 times greater for women aged 50 years and older living with HIV than for those without HIV (95% CI, 1.24 to 8.22, *P* = .016; Table 3). Similarly, women aged 50 years and older with baseline vitamin D insufficiency or deficiency (26.0%) were also more likely to have osteoporosis when accounting for the same covariates (odds ratio [OR], 2.56 [95% CI, 1.01 to 6.54], *P* = .049). By contrast, obesity was associated with reduced odds of having osteoporosis (OR, 0.21 [95% CI, 0.08 to 0.51], *P* = .001). Neither breast cancer receptor status nor elements of the patient's reproductive history (specifically age at menarche/menopause and number of full-term pregnancies) were significantly correlated with osteoporosis in univariable analysis and were thus excluded from the model. Because only eight women in the full cohort had abnormal Z-scores, the logistic regression model for women younger than 50 years was not robust (likelihood ratio chi-square test *P* value .733).

**TABLE 3:** Factors Associated With Osteoporosis in Women Aged 50 Years and Older: Results of Logistic Regression

Variable	Odds Ratio (95% CI)	*P*
HIV infection	3.20 (1.24 to 8.22)	.016
Obesity (BMI ≥30 kg/m^2^)	0.21 (0.08 to 0.51)	.001
Age >65 years	2.96 (1.04 to 8.47)	.042
Grade 3 histology	0.46 (0.19 to 1.15)	.097
Stage III disease	0.93 (0.39 to 2.21)	.875
Vitamin D insufficiency/deficiency	2.56 (1.01 to 6.54)	.049

## DISCUSSION

Our study represents the first analysis, to our knowledge, of BMD among patients with breast cancer before treatment in SSA. Over 60% of the women aged 50 years and older in our cohort had osteopenia or osteoporosis that would typically warrant medical management to reduce further bone losses and the risk of osteoporotic fractures, irrespective of the therapy offered for their breast cancer. We found that age older than 65 years, HIV infection, and vitamin D insufficiency/deficiency were all associated with a significantly increased risk of pretreatment osteoporosis, suggesting that patients meeting these criteria should be more closely monitored for osteoporotic complications both before starting and while on AIs or chemotherapy. Importantly, each of these factors—unlike BMD—can be easily determined in most clinical settings, even in public hospitals where diagnostic resources are limited.

Given the limited access to DXA scanners in SSA, most women with baseline osteopenia or osteoporosis currently start systemic therapy for their breast cancer—including AIs and chemotherapy, which are known to worsen bone health—without their health care providers knowing their pretreatment BMD. As such, they are generally not prescribed bone-protective medications, such as calcium, vitamin D, and bisphosphonates, which have long been proven to reduce the risk of osteoporotic complications.^31^ In these settings and populations, our findings can help optimize existing fracture risk prediction tools such as the FRAX model, which estimates the 10-year probability of fractures and was updated in 2021 to more accurately reflect data from South African ethnic groups.^32^ In particular, our analysis suggests the addition of HIV infection and vitamin D insufficiency/deficiency as risk factors may improve the performance of such predictive models in SSA. Similar tools could be developed to enable more appropriate enrollment of women with breast cancer into interventional trials aimed at reducing the incidence of osteoporotic fractures.

The majority of existing literature on bone health in women with breast cancer has focused on characterizing the impact that breast cancer and its therapy have on BMD and the rate of osteoporotic complications well after patients are diagnosed. The burden of osteopenia and osteoporosis before initiation of cancer therapy has not been as thoroughly examined. One exception was a prospective cohort study of 344 women newly diagnosed with breast cancer in Israel in which DXA scans were performed at the time of diagnosis; this group did not demonstrate a significant difference in the prevalence of osteoporosis compared with healthy controls (11.6% *v* 8.8%, *P* = .332), nor an association between baseline BMD and breast cancer characteristics (grade, stage, and receptor status).^33^ In contrast to our analysis, the authors of that article did not comment on whether other clinical or demographic variables (such as age and BMI) were associated with baseline BMD. Furthermore, the study population was 98.8% Jewish, limiting the generalizability of their results, especially among Black women and those living with HIV.

While our results are most relevant in environments where DXA machines are not widely available, they can also be used to more generally risk stratify women newly diagnosed with breast cancer. Even in high-income countries where access to DXA scanners is robust, the uptake of clinically indicated scans is suboptimal. In a retrospective review of 342 women with breast cancer in the US state of Washington, 56% of the participants had not undergone a DXA scan in the 14 months after starting an AI, with adherence to national guidelines even lower outside the urban region of Seattle.^34^ Furthermore, there are considerable racial disparities, with one study of 2,409 patients with breast cancer in the United States finding that only 33.3% of Black women had a baseline DXA scan before being prescribed an AI, compared with 53.5% of White women (*P* < .001).^35^

The ability to build upon the long-standing prospective SABCHO study was an important strength of our analysis, allowing us to recruit a cohort of women with high rates of HIV from a large academic hospital in Johannesburg. Our multidisciplinary research group has developed deep expertise in enrolling women to the study and longitudinally collecting complete data on cancer outcomes, in addition to baseline demographic and clinical data with few missing values. The fact that we did not conduct a case-control study, however, represents a key limitation, preventing us from directly comparing BMD in our cohort with a matched population of women without breast cancer living in Soweto. Moreover, we did not test for bone turnover biomarkers (BTMs) such as procollagen type I N-propeptide (PNP1) and carboxy-terminal cross-linked telopeptide of type 1 collagen (CTX-1), which some prospective studies have suggested may be helpful in estimating fracture risk, an implicit goal of our research.^36^ BTMs have also been studied extensively in the context of HIV infection and ART use to better understand the mechanisms of bone loss in individuals living with HIV.^37-39^

In conclusion, overall, our study establishes a foundation for understanding the factors associated with osteoporosis—such as HIV, low vitamin D, and age older than 65 years—among women with breast cancer before treatment. We also find that obesity has a protective effect on BMD. Further studies, including follow-up DXA scans on the women in our cohort, will be needed to better understand these associations and then to quantify the impact of HIV status on the rate of AI-induced BMD losses in patients with breast cancer. In addition, it would be valuable to validate the results of our analysis in women of African descent in the United States as well as other communities in SSA. Moving forward, efforts should be made to design interventional studies where women at highest risk of osteoporosis and subsequent fractures are targeted for intensified screening and, if indicated, initiation of bone-modifying therapy.
